# Modifications in Leaf Anatomical Traits of *Coffea* spp. Genotypes Induced by Management × Season Interactions

**DOI:** 10.3390/plants14050828

**Published:** 2025-03-06

**Authors:** Larícia Olária Emerick Silva, Rafael Nunes de Almeida, Rodrigo Barbosa Braga Feitoza, Maura Da Cunha, Fábio Luiz Partelli

**Affiliations:** 1 Centro Universitário do Norte do Espírito Santo, Federal University of Espírito Santo, São Mateus 29932-900, ES, Brazil; 2 Centro de Ciências e Tecnologias Agropecuárias, State University of North Fluminense Darcy Ribeiro, Campos dos Goytacazes 28013-602, RJ, Brazil; 3 Centro Biociências e Biotecnologia, State University of North Fluminense Darcy Ribeiro, Campos dos Goytacazes 28013-602, RJ, Brazil

**Keywords:** *Coffea arabica*, *Coffea canephora*, environmental effect, genetic effect, stomatal density

## Abstract

Leaf anatomical traits are influenced by environmental and genetic factors; however, studies that investigate the genotype × environment interaction on these traits are scarce. This study hypothesized that (1) the leaf anatomy of *Coffea* spp. genotypes is varied, and (2) interactions between managements and seasons significantly influence leaf anatomical traits, inducing a clear adaptation to specific environments. Possible modifications of leaf anatomy in *Coffea* spp. genotypes were investigated under different managements: full-sun monoculture at low-altitude (MLA), full-sun monoculture at high altitude (MHA), and low-altitude agroforestry (AFS), in winter and summer. The genotype influenced all leaf anatomical traits investigated, contributing to 2.3–20.6% of variance. Genotype × environment interactions contributed to 2.3–95.8% of variance to key traits. The effects of genotype × management interactions were more intense than those of genotype × season interactions on traits such as leaf thickness, palisade parenchyma thickness, abaxial epidermis, and polar and equatorial diameter of the stomata. The management AFS was more effective in altering leaf anatomical traits than the altitude differences between MLA and MHA, regardless of the season. These findings provide valuable insights for future research and for the development of strategies to improve the adaptation of coffee plants to changing environmental conditions.

## 1. Introduction

Global coffee production has increased significantly over the past 30 years, reaching nearly 176.2 million 60 kg bags in 2024. While Europe and North America are the largest consumers, South America dominates production, with Brazil leading the ranking of the top five coffee producers [1]. The two most traded coffee species are Arabica (*Coffea arabica*) and Conilon/Robusta (*C. canephora*). Both are grown in regions near the Equator, between the Tropics of Cancer and Capricorn [2]. In the choice of the most adequate coffee type for cultivation, edaphoclimatic requirements have direct influence. Both species are widely produced in Brazil; the optimal temperature and altitude, respectively, are between 18 and 23 °C and 500 and 800 m a.s.l. for Arabica coffee and between 22 and 26 °C and up to 500 m a.s.l. for Conilon/Robusta coffee [3].

Global warming, caused by the increase in average global temperature, has become a cause for concern worldwide, as climate change will lead to a decrease in agricultural production [4]. Coffee production will be negatively impacted, as climate change may reduce suitable areas for coffee cultivation, especially in low-latitude and low-altitude regions [5,6]. Coffee trees are sensitive to climate change, especially temperature and water availability [7]. Global coffee production increased from 4.53 million tons in 1961 to approximately 11 million tons in 2022 [8]. However, this growth was not uniform across regions. While production in Asia surged by more than 1000% over the past 50 years, increases in South America and Africa ranged between 50% and 100% [8]. This disparity can be attributed to factors such as the expansion of consumer markets in Asia and climate change, which has constrained production growth in tropical regions like South America and Africa.

Temperature determines the duration of phenological phases and germination rates [9], and water stress affects several physiological processes such as photosynthesis, growth, and yield of coffee trees [3]. Several studies have tried to predict areas that will in the future be suitable or unsuitable for coffee cultivation due to climate changes [6,10]. However, the identification, selection, and development of coffee cultivars capable of adapting to different environmental conditions is equally relevant [11,12].

Genotype × environment (G × E) interactions, especially complex ones, can hamper the development of new cultivars [13] due to the differential response of a given genotype to biotic or abiotic factors or an interaction thereof in a given environment. Recently, the G × E interactions in coffee plants have been analyzed for traits related to yield [14,15], sensory profile [16], and floral morphology [13]. Some studies have demonstrated genetic variability based on coffee leaf anatomy [17,18]; however, studies evaluating G × E interaction effects on these traits are scarce.

Leaf anatomy is influenced by several environmental factors, e.g., light exposure, water availability, and altitude [19]. These factors directly affect essential physiological processes, such as light distribution within the leaf, CO_2_ diffusion, transpiration, and maintenance of leaf structural integrity [20]. Leaves exposed to intense light (sun leaves) tend to be smaller, thicker, and have a more developed palisade parenchyma than lacunar parenchyma [21,22]. In addition, their stomatal density is higher than that of shade leaves [23,24]. Water availability also plays a crucial role. As an adaptive strategy to water stress, lacunar parenchyma thickness [25] and stomatal closure rates are increased [26], while stomatal density rises and stomatal size shrinks [27]. These anatomical variations are key to understanding how the plant morphology adjusts in response to different environmental conditions, contributing to survival and photosynthetic efficiency.

Leaf anatomy varies along altitudinal gradients due to changes in the partial CO_2_ pressure [28,29]. Although the effect of altitude on leaf anatomy is not yet fully understood, studies indicate that at higher altitudes, the number of epidermal cells and stomatal density may increase [30,31], while the stomatal density and index are reduced [32]. These changes highlight the complexity of G × E interaction effects on leaf anatomical traits. Improving the understanding of this interaction is essential to identify top-performing genotypes under diverse management conditions. This approach represents a promising strategy to increase coffee yields in Brazil, particularly in view of the challenges caused by climate change.

In this context, we hypothesized that (1) the leaf anatomy of *Coffea* spp. genotypes is varied, and (2) interactions between managements and seasons significantly influence leaf anatomical traits, inducing a pronounced adaptation to specific environments. This study investigated possible modifications of leaf anatomical traits in different *Coffea* spp. genotypes grown under different managements in different seasons.

## 2. Results

### Estimation of Genetic Effects and Genotype Classification

**Figure 1:**
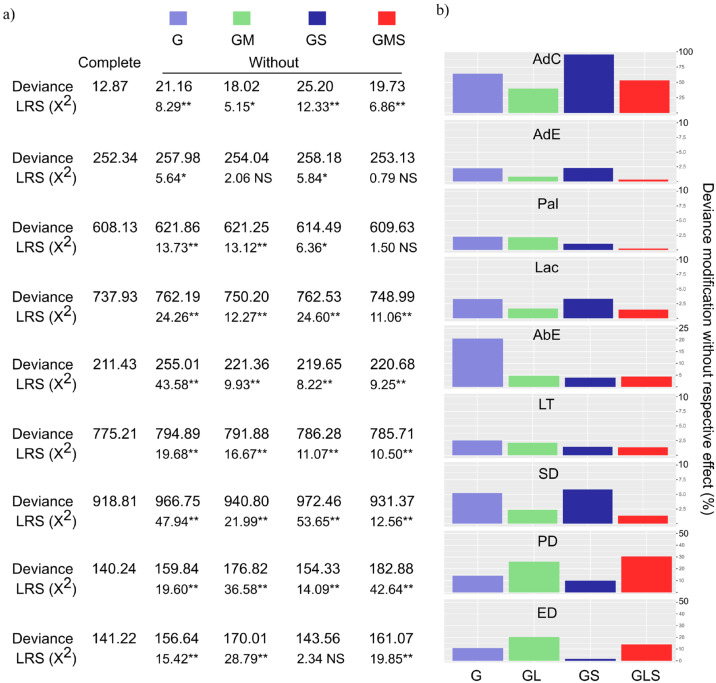
(**a**) Predicted effect of genotype (G) variation sources and interactions with management (M) and seasons (S) in full model for predicting breeding values; (**b**) deviance test for *Coffea* spp. genotypes evaluated under three managements, in two seasons, for leaf anatomical traits: adaxial cuticle (AdC, μm), adaxial epidermis (AdE, μm), palisade parenchyma (Pal, μm), lacunar parenchyma (Lac, μm), abaxial epidermis (AbE, μm), leaf thickness (LT, μm), equatorial diameter (ED, μm), polar diameter (PD, μm), and stomatal density (SD, number of stomata per mm^2^); LRT = difference in deviance values between full model and model without respective source of variation. * and ** indicate significant values at 5 and 1% probability, considering chi-square distribution (X^2^) with one degree of freedom (reference values: 3.84 and 6.83, at 5 and 1% probability, respectively). NS = not significant.

Deviance analysis revealed that genotype (G) variations caused significant effects on all nine evaluated traits (Figure 1a). Only AdE was not affected by the genotype × management (GM) interaction, while the genotype × season (GS) interaction caused no effect on ED. The interaction among genotypes, management, and seasons (GMS) had no significant effect on AdE and Pal (Figure 1a).

**Figure 2:**
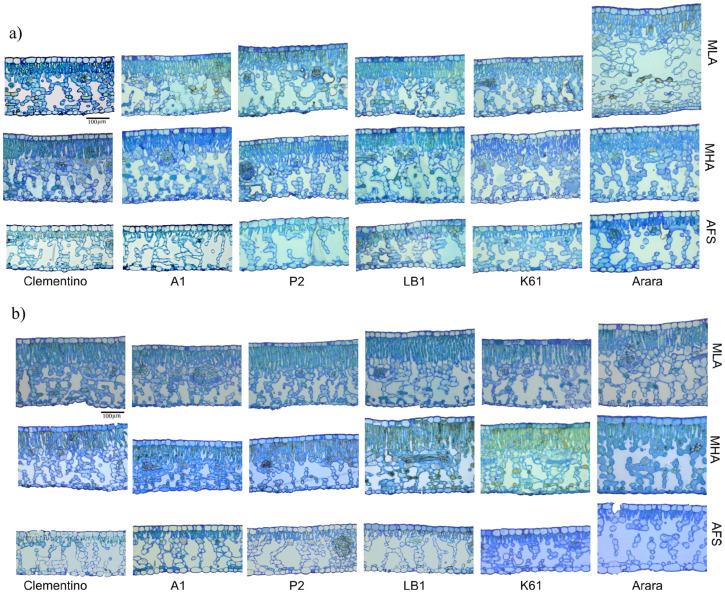
Optical microscopy images of leaf blades of different *Coffea* spp. genotypes evaluated under three managements, in two seasons. (**a**) Summer; (**b**) winter. MLA, full-sun monoculture at low-altitude; MHA, full-sun monoculture at high altitude; AFS, low-altitude agroforestry system.

Regarding the magnitude of effects (Figure 1b), the genotype source of variation caused the greatest modifications in deviance values (2.3–20.6%) when excluded from the model to explain Pal, AbE, and LT; thus, the G variation source was the most influential for this set of traits (Figure 2). The GM interaction had the strongest effect (20.4% of deviance modification) only for ED, whereas the GS interaction was more pronounced for AdC, AdE, Lac, and SD (2.3–95.8%). Conversely, the GMS interaction was most important for PD, causing a deviance modification of 30.4% when excluded from the model (Figure 1b).

The trait heritability (h^2^) was only high for PD (Table 1) while the others had low (<0.40) or very low (<0.20) h^2^ values. In MLAs (full-sun monoculture at low-altitude in summer), the overall means were lowest for AdE and AbE; in AFSw (low-altitude agroforestry system in winter) the means were lowest for AdC, Pal, LS, and SD; and in AFSs (low-altitude agroforestry system in summer), the means were lowest for Spo, PD, and ED (Table 1).

**Figure 3:**
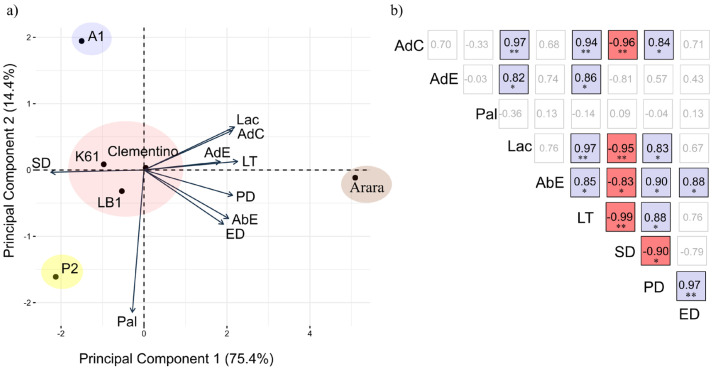
(**a**) Principal component analysis for breeding values of nine leaf anatomical traits evaluated in *Coffea* spp. genotypes under three managements, in two seasons; (**b**) Pearson’s correlation coefficient (blue squares for positive correlations and red squares for negative correlations) between following traits: adaxial cuticle (AdC, μm), adaxial epidermis (AdE, μm), palisade parenchyma (Pal, μm), lacunar parenchyma (Lac, μm), abaxial epidermis (AbE, μm), leaf thickness (LT, μm), equatorial diameter (ED, μm), polar diameter (PD, μm), and stomatal density (SD, number of stomata per mm^2^). * and ** indicate statistically non-zero coefficients by *t*-test at 5 and 1% probability, respectively.

Principal component analysis (PCA) for the nine traits showed the possibility of a simplified grouping, in that the first two components could explain 88.8% of the total variation (Figure 3a). The traits Spo, AdC, AdE, LT, PD, AbE, ED, and SD were correlated with PC 1, SD was negatively related with the others, and PC 2 was predominantly related to Pal.

In the combined analysis of managements and seasons, the genotypes P2 and A1 stood out with a more distant position from the others due to the extreme Pal values, which were highest for P2 and lowest for A1 (Figure 3a). For SD, these two genotypes also had the highest breeding values and low or intermediate values for the remaining traits. Conversely, genotype Arara (*C. arabica*) was isolated from the others with low breeding values for SD and Pal, and high values for all other traits (Figure 3a). In the genotype group with Clementino, K61, and LB1, the breeding values were intermediate to low for all traits, compared with the other genotypes.

The magnitude of the correlations indicated by PCA was estimated using Pearson’s correlation coefficient, indicating strong negative correlations among SD and AdC, Spo, AbE, LT, and PD (Figure 3b). No correlation of Pal with the other traits was detected. AdC was strongly positively correlated with Spo, LT, and PD, while AdE only had a strong positive correlation with Lac and LT. In addition, Lac was strongly positively correlated with LT and PD, while AbE was strongly positively correlated with LT, PD, and ED. Finally, LT was strongly positively correlated with PD, and PD was strongly positively correlated with ED (Figure 3b).

To study contrasts in genotype adaptability and for the formation of mega-environments, the traits Pal, LT, and SD were selected. For having none and negative correlations with the other traits, respectively, Pal and SD were chosen. On the other hand, LT was chosen to represent the remaining traits due to the strong direct correlation with most of them.

**Figure 4:**
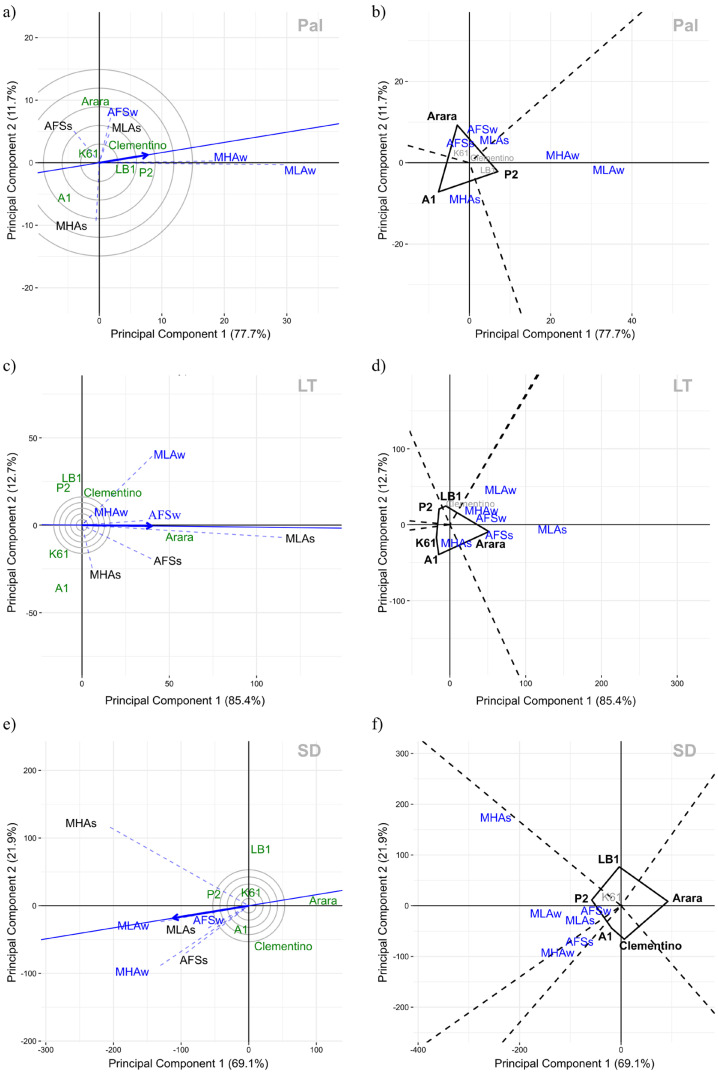
Biplot (GGE-Biplot) to assess discriminating power of environments and adaptability of genotypes expressed in leaf anatomical traits of *Coffea* spp.: (**a**) discriminating power and representativeness of environments for Pal—palisade parenchyma (μm); (**b**) adaptability of genotypes (which-won-where) regarding Pal breeding values (BLUPs); (**c**) discriminating power and representativeness of environments for LT—leaf thickness (μm); (**d**) adaptability of genotypes (who-won-where) regarding LT breeding values; (**e**) discriminating power and representativeness of environments for SD—stomatal density (number of stomata per mm^2^); (**f**) adaptability of genotypes (which-won-where) regarding SD breeding values. MLA—full-sun monoculture at low-altitude; MHA—full-sun monoculture at high altitude; AFS—low-altitude agroforestry system.

An analysis of the G × E interaction (GGE-Biplot) for Pal revealed a corresponding response of the genotypes to the environments AFSw, AFSs, and MLAs, and also between MHAw (full-sun monoculture at high altitude in winter) and MLAw (full-sun monoculture at low-altitude in winter). In MHAs (full-sun monoculture at high altitude in summer), the genotype response was opposite or different from that in the other environments (Figure 4a). In MLAw, the power of genotype discrimination was highest, and in MLA, the greatest representativeness for the set of environments under study was the greatest. These environmental differences revealed the possibility of forming three mega-environments (Figure 4b). In the mega-environment formed by AFSs, AFSw, and MLAs, genotype Arara had the highest Pal values. Genotype P2 stood out in the mega-environment that comprises MHAw and MLAw and genotype A1 in MHAs, with the highest breeding values for Pal (Figure 4b). The GGE Biplot for Pal represented 89.4% of the total variation in the first two components.

In the GGE Biplot analysis for LT, the first two components represented 98.1% of the total variation (Figure 4c). This indicated a positive correlation among all environments and that these correlations were stronger between different environments in the same season (winter or summer). While AFSw was most representative of the set of environments, MLAs had the greatest power of genotype discrimination (Figure 4c). In this single mega-environment that included all environments, genotype Arara stood out with the highest breeding values for LT (Figure 4d).

GGE Biplot analysis for SD represented 71.0% of the total G variation in the environments, indicating a positive correlation among all of them (Figure 4e). Whereas MLAw was most representative of the set of environments under study, MHAs had the greatest power of genotype discrimination. The mega-environment grouping indicated the possibility of joining the environments MHAs, MLAw, MLAs, and AFSw in one group and MHAw and AFSs in another. The genotypes P2 and A1 had the highest breeding value for SD in these mega-environments, respectively (Figure 4f).

Considering genotype stability, the index based on Finlay–Wilkinson regression slopes showed that Pal, K61, and Clementino exhibited higher stability across environments, whereas P2 and LB1 were the most responsive under favorable conditions for an increase in parenchyma thickness (Table 2). A similar result was observed for LT, although P2 tended to show greater stability for LT compared to Pal. As for SD, the most stable genotypes were Clementino and A1, whereas P2 and LB1 were the most responsive under favorable conditions. The *C. arabica* genotype (Arara) showed the lowest responsiveness to environmental variations for all three traits.

## 3. Discussion

The evaluation of six coffee genotypes in six environments (three managements and two seasons) confirmed the influence of the genotype on all leaf anatomical traits analyzed. Previous studies also related differences at the genotype level. Carelli et al. [33] observed variations in Pal, Lac, and mesophyll thickness in six *Coffea* species. In a study on 13 leaf and physiological anatomical traits of 11 *C. arabica* genotypes, Santos et al. [33] identified nine traits with greater relevance for genotype discrimination, including AdE, AbE, Lac, SD, and traits with lower discriminatory power, including AdC, Pal, PD, and ED. Similarly, Alberto et al. [18], in a study with nine *C. arabica* genotypes, reported genotypic differences for the nine leaf anatomical traits investigated in this study, confirming our results.

In this study, the genotypes of two species were investigated: five *C. canephora* genotypes and one *C. arabica* genotype. Differences between the genotypes of the different species were expected, as reported elsewhere [18,33,34]. However, the five evaluated *C. canephora* genotypes were clones that compose commercial cultivars, which indicates that they underwent selection, particularly with regard to yield, plant vigor, and disease resistance [35,36]. This selection process, driven by yield, may indirectly affect other correlated traits, either favorably or unfavorably (negative correlation), even if these are not direct selection targets. In this study, however, the difference in leaf anatomical traits among the *C. canephora* genotypes (which underwent a selection process) show that, for such traits, there was no significant reduction in genetic variability caused by selection for yield, plant vigor, or disease resistance.

Correlations with grain yield were not investigated in this study. As these are young crops (2 to 4 years old), yield data are inconsistent for this type of analysis, since at least five evaluations (harvests) of the same crop are recommended to ensure reliable yield data [37]. Nonetheless, the genotypic variability observed in these genotypes, which have already been selected for high yields, supports the hypothesis of no strong direct correlation between leaf anatomical traits and yield. Yield in *Coffea* is a complex trait, controlled by many genes, and generally without strong correlations with other phenotypic traits, making indirect selection strategies difficult [38]. However, studies that compile yield data of these crops over the next few years may help advance the investigation and quantification of these correlations.

Although somewhat similar research on coffee leaf anatomical traits has been published, the interactions of genotypes under different managements (GM) in different seasons (GS) presented in this study were yet unknown. Considering the degree of modification (deviance) calculated for the full statistical model (Figure 1b), the effects of GM were greater than those of GS interaction for Pal, AbE, LT, PD, and ED. Regarding the overall means for the environments (Table 1), the means of Pal and LT decreased and the mean AbE increased under AFS, both in winter and summer. Additionally, AFS produced no reduction in the overall mean for PD and ED in the summer evaluations. The most marked reduction was recorded for Pal, with a consequent reduction in TL (Table 1). Agroforestry systems of coffee/rubber intercrops reduce light incidence, affecting plant development [39]. We have pointed out that one of the consequences is a reduction in leaf thickness and palisade parenchyma. The palisade parenchyma is an important structure capable of storing chlorophyll pigments and is consequently essential for plant photosynthesis. Investigating the effect of shading on four *C. canephora* genotypes, Assis et al. [40] also found a reduction in Pal and mesophyll thickness under increased shading levels. The authors showed that at higher shade levels, the highest-yielding genotypes were those with the least Pal and Lac reduction, compared to full-sun cultivation.

Considering that the crop yield results consistently followed the literature data, genotype Arara (*C. arabica*) was expected to have the lowest yield reduction under AFS compared with the yield potential under full-sun. The results were the highest Pal and LT values of this genotype in these environments (Figure 4b,d). Although the leaf anatomical structures of *C. canephora* genotypes had greater alterations in shaded environments, Silva Neto et al. [41] reported that management under adequate shading levels can promote gains in beverage quality attributes for this species.

In contrast to the performance of genotype Arara, Morais et al. [42] observed that shaded *C. arabica* plants had thinner cuticles (AdC) and cell walls, mesophyll with less volume, increased intercellular spaces, epidermis with lower stomatal density (SD), and small subsidiary cells, resulting in a lower photosynthetic rate. As our research evaluated only one *C. arabica* genotype, further studies with a greater number of genotypes and managements are needed to investigate the extent of GM interaction between varieties of this species.

**Figure 5:**
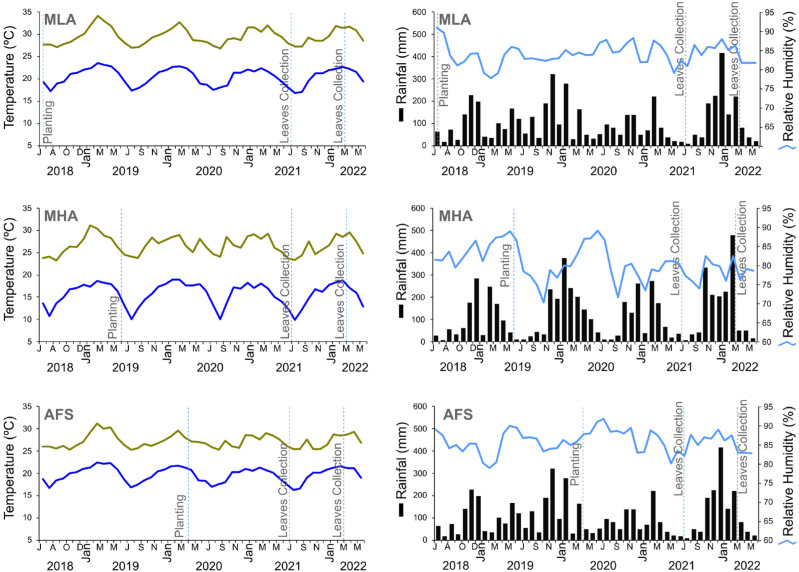
Minimum (dark blue line) and maximum (green line) daily temperature, cumulative monthly rainfall (bars), and relative air humidity (light blue line) from June 2018 to June 2022, under three *Coffea* spp. managements: full-sun monoculture at low-altitude (MLA), full-sun monoculture at high altitude (MHA), and low-altitude agroforestry system (AFS).

Overall, the shading applied in AFS was more effective in causing modifications to leaf anatomical traits of the coffee genotypes than the altitude difference between MLA and MHA in both seasons (Table 1). Apart from the reduced luminosity, the AFS system promoted a reduction in maximum temperatures and a small increase in minimum temperatures and relative humidity (Figure 5). Although the maximum daily temperatures under MHA remained in a range similar to AFS (25–30 °C), the minimum temperatures and relative humidity reached lower levels (some days, around 5 °C in the winter).

Leaf anatomical traits are directly related to plant efficiency in radiation protection, light energy capture, and promoting gas exchange essential to thermal regulation, nutrient transport, and photosynthesis. Therefore, the success of coffee cultivation in agroforestry systems depends on the selection of genotypes better adapted to these conditions [43]. This selection may prove difficult for plant breeders, since the heritability estimates (h^2^) were low for most traits (Table 1). This includes traits less correlated with each other and of greater interest for genotype selection, such as Pal, LT, and SD (Figure 3). Low h^2^ values indicate a predominance of environmental effects [44], represented here by the different managements and seasons, which was well illustrated by the deviance analysis (Figure 1).

The effects of the GS interaction were greater for AdC, AdE, Lac, and SD. The winter (April to September) was characterized by low temperatures, accumulated rainfall, and high relative humidity in the three managements (Figure 5). In the systems MHA and AFS, an increase in AdC, AdE, and SD and a slight decrease in Lac were observed in leaves evaluated in the summer. Under MLA, leaves evaluated in the summer had lower AdC, AdE, and SD values, with a simultaneous increase in Lac. Although the leaves were sampled in different seasons, the climatic variations that occurred during their development (prior to collection) must be taken into account. In this study, the leaves were collected at the third node of the plagiotropic branches in the middle third of the plants. Even when a 12-month period prior to collection—most likely the beginning of leaf development [45]—is taken into consideration, it was observed that the development of leaves collected in the summer began under higher rainfall and higher temperature and humidity than that of leaves collected in the winter (Figure 5). Such modifications across the seasons show clearly that plants adapt the development of their leaf structures throughout growth, according to microclimatic modifications [46]. This adaptation occurs regardless of the management method, as proven by the significant GMS interaction for most traits (aside from AdE and Pal). The GS interaction for these traits also indicated the need for studies in multiple seasons to determine the most appropriate number of seasons that would ensure more accurate inferences about coffee leaf anatomical traits. Evaluating a greater number of seasons would be expected to raise the h^2^ estimates and thus contribute to increasingly accurate inferences.

In the analysis of correlations among traits, no linear correlations with other traits were found for Pal (Figure 3). At the level of breeding values (best linear unbiased predictors—BLUPs), linear correlations suggest high genetic independence for a certain trait in relation to the others. For coffee plants grown on four slopes at different latitudes, positive, albeit weak or moderately weak, correlations (0.2 to 0.5) were found among Pal and LT, AdE, and Lac [47]. Conversely, the strong negative correlation between SD and the other traits (except Pal and AdE) indicated a possible genetic linkage between these traits, since the favorably high SD values disfavored this set of traits. The traits Lac, AdC, AdE, LT, PD, AbE, and ED were strongly correlated with each other, and can be represented by the one with the greatest discrepancy between the genotypes evaluated, and, consequently, with greater power of discrimination. In this study, LT had the greatest discriminatory power and numerous positive correlations with the other traits. A lower number of traits would be interesting to optimize evaluations and make the study of a greater number of genotypes possible [48], since the measurement of these traits for high-resolution image acquisition is highly time- and resource-demanding.

In the evaluations, genotypes Arara, A1, and P2 most frequently had the highest Pal, LT, or SD values in different groups of cropping systems and seasons. These genotypes are suggested for analysis in future studies, especially to address the potential for adaptation to environmental stresses that require greater plant adaptation in terms of photosynthetic efficiency, thermal regulation, and gas exchange intensity [6,10]. The meta-analysis by Poorter et al. [49] shows that several studies reveal that SD plays an important role in the adaptation of C3 plants (such as coffee), especially in environments with different CO_2_ concentrations. Thus, an increase in SD appears to be an important strategy for CO_2_ capture in low-concentration environments, while a decrease in SD promotes reduced CO_2_ diffusion between cells in high-concentration environments [49]. This influence of environmental modifications on stomatal development is reflected in genotype plasticity (Table 2) and reveals the potential effect of epigenetic factors in accelerating plant responses to climate change, as also reported by Kim and Torii [50].

The current scenario of climate change requires constant advances in understanding plant adaptation strategies. This study showed the versatility of plants of the genus *Coffea* for adaptation of leaf anatomical structures to different environments, including managements and seasons. These findings may contribute to the development of future research and to formulate a strategy for the adaptation of coffee plants by promoting stability of cultivation.

## 4. Materials and Methods

### 4.1. Experimental Conditions, Plant Material, and Experimental Design

The study considered six genotypes: five of *C. canephora* characterized as Conilon: LB1 and P2 of cultivar Monte Pascoal [36], A1 and Clementino of cultivar Tributun [35]; the pre-selected clone K61, not yet registered as cultivar; and cultivar Arara, as a representative of *C. arabica*. These six genotypes were cultivated in areas under three distinct managements: full-sun monoculture at low-altitude (MLA), full-sun monoculture at high altitude (MHA), and low-altitude agroforestry system (AFS).

All three managements were tested in the state of Espírito Santo, Brazil. The experiment MLA was planted at 36 m a.s.l. in June 2018 in the municipality of São Mateus (18°40′25″ S, 39°51′23″ W), where the climate is Aw (tropical), according to the Köppen–Geiger classification, with dry winters and rainy summers [51]. The average annual temperature in the region is 24 °C and rainfall is approximately 1370 mm [52]. The soil is classified as Acrisol, with a sandy loam texture [53] and undulating relief [54]. Initially, 30 coffee genotypes were planted in this experimental area, arranged in a randomized complete block design (RCBD) with three replications. Each experimental unit consisted of three plants, with a spacing of 2 m between rows and 1 m between plants. Two orthotropic branches of each plant were considered for evaluations.

The experiment MHA was planted at 1100 m a.s.l. in June 2019 in Venda Nova do Imigrante (20°26′08.21” S, 41°03′58.01′′ W), where the climate is Cfb (temperate or subtropical oceanic climate), according to the Köppen–Geiger classification, with a mild summer, no dry season, and evenly distributed rainfall [51]. The average annual temperature in the region is 19 °C and rainfall is approximately 1420 mm [51]. The soil is classified as a dystrophic Red Yellow Ferralsol [50] with a predominant mountainous relief [53]. Initially, 25 coffee genotypes were planted in RCBD with three replications. Each plot consisted of three plants, with a spacing of 3 m between rows and 0.6 m between plants, and two orthotropic branches of each plant were considered for the evaluations.

The AFS experiment consisted of coffee intercropped with rubber trees (*Hevea brasiliensis*), resulting in an estimated light retention of 75%. Rubber trees were planted at 80 m a.s.l. in October 2012 and coffee in April 2020. This area is also located in São Mateus (18°45′17′′ S, 40°06′26′′ W), in the same climate as MLA, but the soil is a Dystrophic Yellow Ferralsol [53]. Initially, 20 coffee genotypes were planted in RCBD with three replications. The experimental units consisted of five plants and each block was centralized between two rows of rubber trees. Coffee was spaced at 6.5 × 0.5 m and a single orthotropic branch of each plant was considered for the evaluations. Rubber trees were spaced at 6.5 × 2.9 m. Monthly cumulative rainfall, daily minimum and maximum temperature records, and relative air humidity from planting to leaf sampling are listed in Figure 5. The climatic data were recorded at weather stations of the Instituto Nacional de Meteorologia (INMET), in the respective municipalities of the experiments. The data for the AFS were adapted from previous information on temperature and humidity variations [55]. Microclimate environmental data were recorded using HOBO U12 Temp/RH/Light/External Data Loggers, installed 3 m above the coffee crop rows in intercropped systems with rubber trees. To minimize external interference, the devices were placed at the center of the crops. Three loggers were used per row as replicates, collecting data every 10 min.

All coffee management practices in the three experiments were applied according to specific technical guidelines for coffee, namely weed control with herbicide and mechanical mowing, preventive phytosanitary management, Ca import, and fertilization. The amount of mineral fertilizer was computed according to the soil and yield analysis, as described by Paye et al. [56]. Crop requirements and phenological stages were also considered, and around 300–500 kg N ha^−1^, 50–80 kg P_2_O_5_ ha^−1^ and 200–400 kg K_2_O_2_ ha^−1^ were applied in six annual applications. The monoculture experiments were drip-irrigated with hoses laid along the planting rows, 5 cm away from the coffee plant trunk, with emitters spaced at 0.5 m, which released the amount of water equivalent to the evapotranspiration calculated for each of the three experiments.

### 4.2. Leaf Anatomy

Leaves were sampled in all three management systems in the winter (July) and summer (March). Six fully expanded leaves per experimental unit were collected in the morning from the third or fourth node of plagiotropic branches. Leaf fragments (1 cm^2^) were taken from the center of the leaves and fixed in 50% FAA (formaldehyde, glacial acetic acid, and 50% ethanol, 1:1:9) for 48 h. In the laboratory, these fragments were inserted in 1.5 mL Eppendorf tubes containing 0.05 M cacodyl buffer, 4% paraformaldehyde, 2.5% glutaraldehyde, and distilled water [57]. For anatomical analyses, the fragments were divided into two subsamples of 0.5 cm^2^ that were washed thrice in 0.05 M cacodyl buffer, at 15 min intervals between washings.

Subsequently, the samples were dehydrated in a series of increasing ethanol concentrations and three times in 100% alcohol, with 1 h intervals between each step. After dehydration, the samples were progressively infiltrated in historesin/ethanol at the following proportions, 1:3, 1:2, 1:1, 2:1, 3:1, and pure historesin, at 48 h intervals. To improve resin penetration, the samples were subjected to a vacuum pressure of −15 to −20 atm for 5 min during historesin infiltration. Subsequently, they were embedded in historesin containing hardener (resin hardener) and left to stand for 48 h for polymerization. After inclusion, the samples were removed from the molds and fixed to 2 cm^2^ wooden supports to ensure stability during cutting.

A manual rotary microtome, Leica RM 2155 (Leica, Nussloch, Germany), was used to cut 4 µm sections of each subsample. The sections were mounted on semi-permanent slides and stained with toluidine blue. In the epidermis dissociation process for visualization of the stomata, the leaf fragments were dipped in Jeffrey’s solution (10% chromic acid + 10% nitric acid, 1:1) [58] for 24 h and then washed in distilled water and 50% ethanol. Subsequently, they were stained with alcohol–safranin and mounted on semi-permanent slides. These slides were examined and documented under a light microscope (Zeiss Axioplan model, White Plains, NY, USA) and photographed with an attached camera (PowerShot A640, CANON, Melville, NY, USA).

The following leaf anatomical traits were recorded: adaxial cuticle thickness (AdC, μm), adaxial epidermis thickness (AdE, μm), palisade parenchymal thickness (Pal, μm), lacunar parenchymal thickness (Lac, μm), abaxial epidermal thickness (AbE, μm) and leaf thickness (LT, μm), equatorial diameter of the stomata (ED, μm), polar diameter of the stomata (PD, μm), and stomatal density (SD, number of stomata per mm^2^). To measure AdC, AdE, Pal, Spo, AbE, LT, ED, and PD, 100× magnification was used and 50× to assess SD. All images were analyzed using ImageJ software (version 1.53t, Wayne Rasband/NIH, Bethesda, MD, USA).

### 4.3. Statistical Analysis

The effects of genotype and G × E interactions (managements and seasons) were estimated by mixed modeling with restricted maximum likelihood estimators (REML), according to the randomized complete block design, at multiple sites and in multiple seasons.

(1) y=Xf+Zg+Qa+Wt+e

where *y* is the data vector (phenotype); *f* is the repetition effect vector of the combinations within managements and seasons, assumed as fixed and added to the overall mean; *g* is the genotypic effect vector, assumed as a random value; *a* is the interaction effect vector between genotypes and seasons, assumed to be random; *i* is the interaction effect vector between genotypes and management, assumed to be random; *t* is the triple interaction vector among genotypes, managements, and seasons, assumed as random; *e* is the error vector (random effect); and *X*, *Z*, *Q*, *T*, and W represent the incidence matrices for the respective effects. From this analysis, heritability (h^2^) was estimated as a genetic parameter for each trait. Based on the REML estimates, the breeding value for each genotype, expressed by the best linear unbiased predictor (BLUP), was also calculated. Deviance analysis inferred the significance of effects for each variation source, based on the difference between deviances from the full model and the model without the effect (LRT), tested by the chi-square method (X^2^), at a significance level of 5%.

The individual genotype BLUP values of each of the six environments (three managements and two seasons) were entered into a double-entry spreadsheet, in which genotypes and environments were considered as independent and the nine traits as dependent factors. After standardization, these data were used for principal component analysis (PCA). Additionally, Pearson’s correlation coefficients between pairs of traits were estimated and tested by the *t*-test (*p* < 0.05).

The BLUP values for Pal, LT, and SD were also entered into new double-entry spreadsheets, one for each trait, where the genotype was considered as independent and the environments as dependent factors. These data were also analyzed by PCA, adapted for the analysis of G × E interaction (GGE-Biplot), as proposed by Yan et al. [59]. The stability indices based on Finlay–Wilkinson regression slopes for Pal, LT, and SD were obtained by regressing the genotypes against the mean genotype value in each environment. The genotype stability expressions in the environments are indicated by the values of b_i_ = 1, bi < 1, and bi > 1, which represent average, high, and low stabilities, respectively. The regression coefficient (b_i_) was calculated using the following estimator:

$$b_{i}=\frac{\sum_{j=1}^{q}\left({\bar{X}}_{ij}-{\bar{X}}_{i.}\right)\left({\bar{X}}_{j.}-{\bar{X}}_{..}\right)}{\sum_{j=1}^{q}{\left({\bar{X}}_{j.}-{\bar{X}}_{..}\right)}^{2}}$$

where *b_i_* is the i-th genotype regression coefficient, *X_ij_* is the mean value of the i-th genotype in the j-th environment, *X_i_*_._ is the mean value of the i-th genotype, *X_j_*_._ is the mean value of the j-th environment, *X*_.._ is the mean value of all environmental indices, and *q* is the number of environments. For REML/BLUP analysis, software Selegen REML/BLUP (version 2020-december) was used [60]. For principal component analysis, stability indices, and graph plotting, software R, version 4.4.1 [61] was used, with the packages metan, GGEBiplot-Gui, factoextra, ggplot2, and psych.

## 5. Conclusions

Seasonal variations and different managements at different locations significantly influenced the leaf anatomical traits of *Coffea* spp. genotypes. Differences in shade level had a greater impact on the performance of the genotypes than differences in altitude. In coffee plants shaded by rubber trees, the palisade and lacunar parenchyma thicknesses of the leaves were thinner, leaf thickness and stomatal density were reduced, and the adaxial and abaxial epidermis thicknesses increased. The leaves collected in winter from a shaded environment had a thinner adaxial cuticle, whereas those collected in the summer had a thickened cuticle. Cultivar Arara (*C. arabica*) had greater palisade parenchyma thickness and, consequently, greater leaf thickness in shaded and low-altitude environments (full-sun), whereas the stomatal density of genotype P2 (*C. canephora*) was highest in all environments. *Coffea* spp. plants adjust leaf anatomical structures in response to different environments, defined by managements and seasons. The development of coffee cultivars capable of adapting to different environmental conditions will make the implementation of more sustainable cultivation systems possible by planting higher-yielding genotypes for the respective specific conditions.

## Figures and Tables

**Table 1:** Heritability estimates with respective standard deviation (sd) and overall mean for leaf anatomical traits in *Coffea* spp. genotypes under three managements, in two seasons. Adaxial cuticle (AdC, μm), adaxial epidermis (AdE, μm), palisade parenchyma (Pal, μm), spongy parenchyma (Spo, μm), abaxial epidermis (AbE, μm), leaf thickness (LT, μm), equatorial diameter (ED, μm), polar diameter (PD, μm), and stomatal density (SD, number of stomata per mm^2^). MLA, full-sun monoculture at low-altitude; MHA, full-sun monoculture at high altitude; AFS, low-altitude agroforestry system.

Traits	h^2^ ± sd	MLAw	MHAw	AFSw	MLAs	MHAs	AFSs	Overall
AdC	0.20 ± 0.12	3.75	3.61	3.39	3.42	4.50	4.40	3.84
AdE	0.01 ± 0.02	19.39	21.08	23.34	18.93	23.84	24.71	21.88
Pal	0.10 ± 0.01	50.91	70.21	25.46	87.23	76.55	32.97	57.22
Lac	0.21 ± 0.11	171.94	182.84	144.03	183.91	162.75	142.68	164.69
AbE	0.12 ± 0.09	17.44	17.31	20.07	15.83	18.42	21.73	18.47
LT	0.10 ± 0.08	263.42	295.04	216.28	309.33	286.06	226.49	266.10
SD	0.37 ± 0.15	374.01	319.29	161.56	302.79	334.56	165.07	276.21
PD	0.75 ± 0.22	19.51	18.80	19.09	19.17	18.86	17.32	18.79
ED	0.31 ± 0.14	13.20	11.90	12.04	11.00	11.61	9.00	11.46

**Table 2:** Genotype stability index according to Finlay–Wilkinson regression slopes for Pal—palisade parenchyma (μm); LT—leaf thickness (μm); and SD—stomatal density (number of stomata per mm^2^) based on genetic values (BLUP).

Genotype	Pal		LT		SD	
	Intercept	Stability	Intercept	Stability	Intercept	Stability
Clementino	−3.10	1.06	−14.32	1.06	18.68	0.94
A1	6.20	0.79	29.64	0.85	52.12	0.92
P2	−7.12	1.21	−38.65	1.10	−87.24	1.49
LB1	−3.95	1.11	−51.16	1.18	−40.23	1.11
K61	−0.90	0.99	−18.51	1.03	50.67	0.89
Arara	8.99	0.82	93.00	0.78	5.99	0.63

## Data Availability

Data is contained within the article.

## References

[1] (2024). ICO-International Coffee Organization-Monthly Coffee Market Report. https://www.icocoffee.org/documents/cy2024-25/cmr-1124-e.pdf.

[2] Nadaf S.A., Shivaprasad P., Babou C., Hariyappa N., Chandrashekar N., Kumari P., Sowmya P.R., Harresh S.B., Rajib P.N., Nagaraja J.S. (2024). Coffee (*Coffea* spp.). Soil Health Management for Plantation Crops: Recent Advances and New Paradigms.

[3] DaMatta F.M., Ramalho J.C. (2006). Impacts of drought and temperature stress on coffee physiology and production: A review. Braz. J. Plant Physiol..

[4] Hassan W., Nayak M.A., Azam M.F. (2024). Intensifying spatially compound heatwaves: Global implications to crop production and human population. Sci. Total Environ..

[5] Bunn C., Läderach P., Ovalle Rivera O., Kirschke D. (2015). A bitter cup: Climate change profile of global production of Arabica and Robusta coffee. Clim. Change.

[6] Lorençone P.A., Oliveira Aparecido L.E., Lorençone J.A., Botega G.T., Lima R.F., Souza Rolim G. (2023). Climate change and its consequences on the climatic zoning of *Coffea canephora* in Brazil. Environ. Dev. Sustain..

[7] Piedra-Bonilla E.B., Cunha D.A., Braga M.J. (2020). Climate variability and crop diversification in Brazil: An ordered probit analysis. J. Clean. Prod..

[8] FAOSTAT (2023). Food and Agriculture Organization of the United Nations. Coffee Production by Region, 1961 to 2022. https://ourworldindata.org/grapher/coffee-production-by-region.

[9] López M.E., Santos I.S., Oliveira R.R., Lima A.A., Cardon C.H., Chalfun-Junior A. (2021). An overview of the endogenous and environmental factors related to the *Coffea arabica* flowering process. Beverage Plant Res..

[10] González-Orozco C.E., Porcel M., Byrareddy V.M., Rahn E., Cardona W.A., Velandia D.A.S., Carrilo G.A., Kath J. (2024). Preparing Colombian coffee production for climate change: Integrated spatial modelling to identify potential robusta coffee (*Coffea canephora* P.) growing areas. Clim. Change.

[11] Martinez H.E., Souza B.P., Caixeta E.T., Carvalho F.P., Clemente J.M. (2020). Water stress changes nitrate uptake and expression of some nitrogen related genes in coffee-plants (*Coffea arabica* L.). Sci. Hortic..

[12] Santos C.S., Freitas A., Silva G.H.B., Pennacchi J.P., Carvalho M.A.F., Santos M.D.O., Moraes T.S.J., Abrahão J.C.R., Pereira A.A., Carvalho G.R. (2023). Phenotypic plasticity index as a strategy for selecting water-stress-adapted coffee genotypes. Plants.

[13] Silva L.O.E., Rodrigues M.J.L., Ferreira M.F.S., Almeida R.N., Ramalho J.C., Rakocevic M., Partelli F.L. (2024). Modifications in floral morphology of *Coffea* spp. genotypes at two distinct elevations. Flora.

[14] Akpertey A., Anim E., Adu-Gyamfi P.K.K., Dadzie A.M., Nyadanu D., Ofori D. (2023). Exploring genotype x environment interaction in Robusta coffee for growth and yield stability under tropical environments. J. Crop Sci. Biotechnol..

[15] Gebreselassie H., Tesfaye B., Gedebo A., Tolessa K. (2024). Genotype by environment interaction and stability analysis using AMMI and GGE-biplot models for yield of Arabica coffee genotypes in south Ethiopia. J. Crop Sci. Biotechnol..

[16] Souza C.A., Rocha R.B., Teixeira A.L., Alves E.A., Espindula M.C. (2021). Genotype-environment interaction for the sensory profile of *Coffea arabica* lines in high temperature regions in the Western Amazon. Genet. Mol. Res..

[17] Araújo L.F.B., Espindula M.C., Rocha R.B., Torres J.D., Campanharo M., Pego W.F.O., Rosa S.D.S. (2021). Genetic divergence based on leaf vegetative and anatomical traits of *Coffea canephora* clones. Sem. Ciênc. Agrár..

[18] Alberto N.J., Ferreira A., Ribeiro-Barros A.I., Aoyama E.M., Silva L.O.E., Rakocevic M., Partelli F.L. (2024). Plant morphological and leaf anatomical traits in *Coffea arabica* L. cultivars cropped in Gorongosa Mountain, Mozambique. Horticulturae.

[19] Dorken V.M., Lepetit B. (2018). Morpho-anatomical and physiological differences between sun and shade leaves in *Abies alba* Mill. (Pinaceae, Coniferales): A combined approach. Plant Cell Environ..

[20] Tholen D., Boom C., Zhu X.G. (2018). Opinion: Prospects for improving photosynthesis by altering leaf anatomy. Plant Sci..

[21] Kim G.T., Yano S., Kozuka T., Tsukaya H. (2005). Photomorphogenesis of leaves: Shade-avoidance and differentiation of sun and shade leaves. Photochem. Photobiol. Sci..

[22] Hoshino R., Yoshida Y., Tsukaya H. (2019). Multiple steps of leaf thickening during sun-leaf formation in Arabidopsis. Plant J..

[23] Boardman N.T. (1977). Comparative photosynthesis of sun and shade plants. Ann. Rev. Plant Physiol..

[24] Singla A., Sharma R., Chhabra R., Vij L., Singh P. (2021). Effect of varying shade intensities of green net on growth and stomatal attributes of different Ocimum species. Proc. Natl. Acad. Sci. India Sect. B Biol. Sci..

[25] Baroni D.F., Souza G.A., Bernado W.D.P., Santos A.R., Barcellos L.C.D.S., Barcelos L.F., Correia L.Z., Almeida C.M., Verdim Filho A.C., Rodrigues W.P. (2024). Stomatal and non-stomatal leaf responses during two sequential water stress cycles in young *Coffea canephora* plants. Stresses.

[26] Avila R.T., Cardoso A.A., Almeida W.L., Costa L.C., Machado K.L., Barbosa M.L., DaMatta F.M. (2020). Coffee plants respond to drought and elevated [CO_2_] through changes in stomatal function, plant hydraulic conductance, and aquaporin expression. Environ. Exper. Bot..

[27] Caine R.S., Harriso E.L., Sloan J., Flis P.M., Fischer S., Khan M.S., Nguyen P.T., Nguyen L.T., Gray J.E., Croft H. (2023). The influences of stomatal size and density on rice abiotic stress resilience. New Phytol..

[28] Korner C., Allison A., Hilscher H. (1983). Altitudinal variation of leaf diffusive conductance and leaf anatomy in heliophytes of montane New Guinea and their interrelation with microclimate. Flora.

[29] Hu J.J., Xing Y.W., Su T., Huang Y.J., Zhou Z.K. (2019). Stomatal frequency of *Quercus glauca* from three material sources shows the same inverse response to atmospheric CO_2_. Ann. Bot..

[30] Wang R., Yu G., He N., Wang Q., Xia F., Zhao N., Xu Z., Ge J., Li C. (2014). Elevation-related variation in leaf stomatal traits as a function of plant functional type: Evidence from Changbai Mountain, China. PLoS ONE.

[31] Rahman I.U., Afzal A., Calixto E.S., Iqbal Z., Abdalla M., Alsamadany H., Parvez R., Romman M., Ali N., Sakhi S. (2022). Species-specific and altitude-related variations in stomatal features of *Berberis lycium* Royle and *B. parkeriana* CK Schneid. Bot. Lett..

[32] Zhang L., Zhang S., Li Q., Quan C. (2020). Reduced stomatal frequency with rising elevation for *Kobresia royleana* on the Tibetan Plateau. Glob. Ecol. Conserv..

[33] Carelli M.L.C., Queiroz-Voltan R.B., Fahl J.I., Trivelin P.C.O. (2003). Leaf anatomy and carbon isotope composition in *Coffea* species related to photosynthetic pathway. Braz. J. Plant Physiol..

[34] Santos C.S.D., Matos N.M.S.D., Rezende T.T., Mauri J., Rodrigues G.C., Veiga A.D., Bartholo G.F., Carvalho M.A.D.F. (2022). Agronomic, anatomic and physiological characterization of *Coffea arabica* L. genotypes on irrigated system in the Central Cerrado. Coffee Sci..

[35] Partelli F.L., Giles J.A.D., Oliosi G., Covre A.M., Ferriera A., Rodrigues V.M. (2020). Tributun: A coffee cultivar developed in partnership with farmers. Crop Breed. Appl. Biotechnol..

[36] Partelli F.L., Covre A.M., Oliosi G., Covre D.T. (2021). Monte Pascoal: First clonal conilon coffee cultivar for southern Bahia-Brazil. Funct. Plant Breed. J..

[37] Santin M.R., Coelho M.C., Sayd R.M., Peixoto J.R., Amabile R.F. (2019). Yield, maturation cycle, and estimates of genetic parameters of Robusta coffee genotypes under irrigation in the Cerrado. Crop Breed. Appl. Biotechnol..

[38] Ferrão R.G., Ferreira A., Cruz C.D., Cecon P.R., Ferrão M.A.G., Fonseca A.F.A., Carneiro P.C.S., Silva M.F. (2008). Inter-trait relations for direct and indirect selection in coffee. Crop Breed. Appl. Biotechnol..

[39] Partelli F.L., Araújo A.V., Vieira H.D., Dias J.R.M., Menezes L.F.T.D., Ramalho J.C. (2014). Microclimate and development of ’Conilon’ coffee intercropped with rubber trees. Pesq. Agropec. Bras..

[40] Assis B.D.P., Gross E., Pereira N.E., Mielke M.S., Júnior G.A.G. (2019). Growth response of four Conilon coffee varieties (*Coffea canephora* Pierre ex A. Froehner) to different shading levels. J. Agric. Sci..

[41] Silva Neto F.J.D., Morinigo K.P.G., Guimarães N.D.F., Gallo A.D.S., Souza M.D.B.D., Stolf R., Fontanetti A. (2018). Shade trees spatial distribution and its effect on grains and beverage quality of shaded coffee trees. J. Food Qual..

[42] Morais H., Medri M.E., Marur C.J., Caramori P.H., Ribeiro A.M.D.A., Gomes J.C. (2004). Modifications on leaf anatomy of *Coffea arabica* caused by shade of pigeonpea (*Cajanus cajan*). Braz. Arch. Biol. Technol..

[43] Terashima I., Hanba Y.T., Tholen D., Niinemets U. (2011). Leaf functional anatomy in relation to photosynthesis. Plant Physiol..

[44] Cruz C.D., Carneiro P.C.S., Regazzi A.J. (2014). Modelos Biométricos Aplicados ao Melhoramento Genético.

[45] Camargo Â.P.D., Camargo M.B.P.D. (2011). Definição e esquematização das fases fenológicas do cafeeiro arábica nas condições tropicais do Brasil. Bragantia.

[46] Rodrigues W.P., Silva J.R., Ferreira L.S., Machado Filho J.A., Figueiredo F.A., Ferraz T.M., Bernado W.P., Bezerra L.B.S., Pereira D., Cespon L. (2018). Stomatal and photochemical limitations of photosynthesis in coffee (*Coffea* spp.) plants subjected to elevated temperatures. Crop Pastu. Sci..

[47] Pérez-Molina J.P., Toledo Picoli E.A., Oliveira L.A., Silva B., Souza G.A., Santos Rufino J.L., Pereira A.A., Ribeiro M.F., Malvicini G.L., Turello L. (2021). Treasured exceptions: Association of morphoanatomical leaf traits with cup quality of *Coffea arabica* L. cv. “Catuaí”. Food Res. Int..

[48] Silva L.O.E., Rodrigues M.J.L., Almeida R.N., Semedo J.N., Rakocevic M., Partelli F.L. (2024). Towards a minimum number of key flower traits in studies of *Coffea* spp. phenotype variability. Sci. Hortic..

[49] Poorter H., Knopf O., Wright I.J., Temme A.A., Hogewoning S.W., Graf A., Cernusak L.A., Pons T.L. (2022). A meta-analysis of responses of C3 plants to atmospheric CO_2_: Dose–response curves for 85 traits ranging from the molecular to the whole-plant level. New Phytol..

[50] Kim E.D., Torii K.U. (2024). Stomatal cell fate commitment via transcriptional and epigenetic control: Timing is crucial. Plant Cell Environ..

[51] Alvares C.A., Stape J.L., Sentelhas P.C., Gonçalves J.L.M., Spavore G. (2013). Köppen’s climate classification map for Brazil. Meteorol. Z..

[52] Instituto Nacional de Meteorologia-INMET Dados das Estações Meteorológicas A616–São Mateus, ES, Brasil e A633-Venda Nova do Imigrante, ES, Brasil. https://mapas.inmet.gov.br/.

[53] Iuss Working Group Wrb (2025). World Reference Base for Soil Resources 2014: International Soil Classification System for Naming Soils and Creating Legends for Soil Maps: Update, 2015.

[54] Santos H.G., Jacomine P.K.T., Anjos L.H.C., Oliveira V.A., Lumbreras J.F., Coelho M.R., Almeida J.A., Araujo Filho J.C., Oliveira J.B., Cunha T.J.F. (2018). Brazilian Soil Classification System.

[55] Araújo A.V., Partelli F.L., Oliosi G., Pezzopane J.R.M. (2016). Microclimate, development and productivity of robusta coffee shaded by rubber trees and at full sun. Rev. Ciênc. Agron..

[56] Paye H.S., Partelli F.L., Martins A.G., Siebeneichler E.A., Partelli F.L., Espindula M.C. (2019). Recomendação de adubação e calagem. CAFÉ CONILON: Conhecimento Para Superar Desafios.

[57] Klein D.E., Moreira G.V., Silva-Neto S.J., Da Cunha M. (2004). The structure of colleters in several species of *Simira* (Rubiaceae). Ann. Bot..

[58] Kraus J.E., Arduin M. (1997). Manual Básico de Métodos em Morfologia Vegetal.

[59] Yan W., Hunt L.A., Sheng Q., Szlavnics Z. (2000). Cultivar evaluation and mega-environment investigation based on the GGE biplot. Crop Sci..

[60] Resende M.D.V. (2016). Software Selegen-REML/BLUP: A useful tool for plant breeding. Crop Breed. Appl. Biotechnol..

[61] R Core Team (2021). R: A Language and Environment for Statistical Computing.

